# Identification of a Novel Inhibitor against Middle East Respiratory Syndrome Coronavirus

**DOI:** 10.3390/v9090255

**Published:** 2017-09-14

**Authors:** Yaping Sun, Huaidong Zhang, Jian Shi, Zhe Zhang, Rui Gong

**Affiliations:** 1 Center for Emerging Infectious Diseases, CAS Key Laboratory of Special Pathogens and Biosafety, Wuhan Institute of Virology, Chinese Academy of Sciences, No. 44 Xiao Hong Shan, Wuhan 430071, China; sunypxw@163.com (Y.S.); hdzhang@wh.iov.cn (H.Z.); shijian@wh.iov.cn (J.S.); zhangzhe@wh.iov.cn (Z.Z.); 2 University of Chinese Academy of Sciences, Beijing 100049, China

**Keywords:** MERS-CoV, fusion inhibitor, six-helix bundle, heptad repeat, five-helix bundle

## Abstract

The Middle East respiratory syndrome coronavirus (MERS-CoV) was first isolated in 2012, and circulated worldwide with high mortality. The continual outbreaks of MERS-CoV highlight the importance of developing antiviral therapeutics. Here, we rationally designed a novel fusion inhibitor named MERS-five-helix bundle (MERS-5HB) derived from the six-helix bundle (MERS-6HB) which was formed by the process of membrane fusion. MERS-5HB consists of three copies of heptad repeat 1 (HR1) and two copies of heptad repeat 2 (HR2) while MERS-6HB includes three copies each of HR1 and HR2. As it lacks one HR2, MERS-5HB was expected to interact with viral HR2 to interrupt the fusion step. What we found was that MERS-5HB could bind to HR2P, a peptide derived from HR2, with a strong affinity value (*K*_D_) of up to 0.24 nM. Subsequent assays indicated that MERS-5HB could inhibit pseudotyped MERS-CoV entry effectively with 50% inhibitory concentration (IC_50_) of about 1 μM. In addition, MERS-5HB significantly inhibited spike (S) glycoprotein-mediated syncytial formation in a dose-dependent manner. Further biophysical characterization showed that MERS-5HB was a thermo-stable α-helical secondary structure. The inhibitory potency of MERS-5HB may provide an attractive basis for identification of a novel inhibitor against MERS-CoV, as a potential antiviral agent.

## 1. Introduction

The newly emerging pathogen Middle East respiratory syndrome coronavirus (MERS-CoV) was first recognized in Saudi Arabia in June 2012 [1]. MERS-CoV causes severe respiratory disease in the lower respiratory tract and is often accompanied by renal failure, showing similar clinical, epidemiological, and virological features to severe acute respiratory syndrome coronavirus (SARS-CoV) which widely spread in China in 2003 [2,3,4,5,6]. As of June 2017, the World Health Organization (WHO) has been notified of 2037 laboratory-confirmed cases of infection with MERS-CoV and at least 710 deaths related to MERS-CoV, showing a much higher fatality rate (about 35%) [7] compared to SARS-CoV (less than 10%) [2,3]. Since September 2012, 27 countries have reported cases of MERS-CoV globally while most cases were found in Saudi Arabia [8,9]. Various therapeutics including peptide inhibitors, neutralizing antibodies, and vaccines are under development for the treatment and prevention of MERS-CoV [10,11,12,13,14,15]. However, there are still no licensed vaccines or antiviral agents against MERS-CoV approved for human use. Therefore, development of efficient countermeasures to curb the spread of MERS-CoV is of great importance.

MERS-CoV is an enveloped positive-sense single-stranded RNA virus and belongs to the lineage C betacoronavirus in the family *Coronaviridae* [16]. Its spike glycoprotein (S) is a class I membrane fusion protein, and contains a surface subunit (S1) and a transmembrane subunit (S2). S1 is in charge of receptor binding, while S2 mediates membrane fusion. MERS-CoV initiates infection through the interaction between the receptor-binding domain (RBD) in S1 and the receptor dipeptidyl peptidase-4 (DPP4, also known as CD26) [17,18,19,20]. Then, S2 changes conformation by inserting its N-terminal fusion peptide into the host cell membrane, after which three heptad repeat 1 (HR1)segments and three heptad repeat 2 (HR2) segments form a six-helix bundle (MERS-6HB) fusion core [10,11]. The three HR1s constitute the central homotrimeric core, and the three HR2s surround the core at the HR1 side grooves in an antiparallel manner. This strong interaction of HR1s and HR2s brings the viral and cell membranes close to each other which triggers fusion. Disrupting the process of MERS-6HB formation can inhibit cell–cell fusion, which leads to abortion of the viral infection. Based on this mechanism, several peptide inhibitors against MERS-CoV and SARS-CoV derived from HR2 have been identified, such as P1 [10], HR2P [11] and CP-1 [21], which prevent the formation of the fusion core by competitively binding to HR1 and blocking the native interaction between HR1 and HR2. Hence, we have put forward an idea that the blockage of fusion core formation could also be achieved through binding to native HR2 by a foreign inhibitor. Therefore, we designed a potential inhibitor named MERS-five-helix bundle (MERS-5HB) including three HR1s and two HR2s. Since MERS-5HB lacks one HR2 segment when compared to MERS-6HB, it is intended to bind with native HR2 in S2 and prevent the formation of the fusion core. A series of experiments were performed to verify our rational design.

## 2. Materials and Methods

### 2.1. Design, Expression and Purification of MERS-5HB

In accordance with the two predicted heptad repeat regions of MERS-CoV S protein (AFS88936.1) [11] from the Learn coil-VMF website [22] and the solved crystal structures of MERS-6HB [10,11], we used the sequences of HR1 from amino acid 984 to 1062 and HR2 from amino acid 1245 to 1289 for our construction. The gene of MERS-5HB was synthesized as a codon of HR1–SGGRGG–HR2–GGSGGSGG–HR1–SGGRGG–HR2–GGSGGSGG–HR1 and subcloned into the vector pET-28a(+) (Invitrogen, Carlsbad, CA, USA) for expression in the *Escherichia coli* strain BL21 (DE3) (Invitrogen). The bacteria were grown at 37 °C in 2× to an optical density (OD_600_) of 0.8 and induced with 1 mM isopropyl-β-d-thiogalactopyranoside (IPTG) at 22 °C for 8 h. The cells were harvested and re-suspended in phosphate buffer saline (PBS) containing 500 mM NaCl, then lysed with 1% Triton X-100 by ultrasonication on ice. The lysate was clarified by centrifugation and the supernatant was passed over a nickel-nitrilotriacetic acid (Ni-NTA) Resin (Qiagen, Hilden, Germany) column pre-equilibrated with PBS containing 500 mM NaCl. The protein was eluted with different concentrations of imidazole. Purified protein was concentrated to a proper concentration determined by the experimental requirements and stored at −80 °C for further analysis.

### 2.2. Binding Assay by Enzyme-Linked Immunosorbent Assay (ELISA)

In order to determine the interaction between MERS-5HB and HR2, we synthesized a biotin-labeled peptide MERS-HR2P representative of HR2 by the standard solid phase peptide synthesis method with purity above 95% (ChinaPeptides, Shanghai, China).

As reported previously [11], the sequence of synthesized MERS-HR2P is Biotin-GGGSLTQINTTLLDLTYEMLSLQQVVKALNESYIDLKEL. An irrelevant peptide HIV-HR2P derived from HR2 in gp41 was used as control. We tested and compared the reactivity by ELISA in two ways which are described as follows.

#### 2.2.1. Binding of MERS-5HB to Immobilized Peptide

A 96-well half-area plate (Costar, New York, NY, USA) was coated with 100 ng/well (50 μL) of Streptavidin (Sangon Biotech, Shanghai, China) in PBS at 4 °C overnight. After blocking with 3% non-fat milk, biotin-labeled MERS-HR2P and HIV-HR2P (100 ng/well) were pipetted into each well for 1 h of incubation at 37 °C. For the binding assay, MERS-5HB (in a 3-fold dilution, with highest concentration of 0.1 μM) was added and incubated for 2 h. Bound MERS-5HB was detected by horseradish peroxidase (HRP)-labeled 6× His Monoclonal Antibody (Proteintech, Wuhan, China). The absorbance was read at 405 nm with a Synergy 2 Multi-Mode Reader (BioTek, Winooski, VT, USA).

#### 2.2.2. Binding of Peptide to Coated MERS-5HB

A microtiter plate was coated with 100 ng/well (50 μL) of MERS-5HB in PBS at 4 °C overnight. After blocking, serially diluted MERS-HR2P and HIV-HR2P were added and incubated for 1 h. The initial concentration was 1 μM and the subsequent was diluted by a 5-fold gradient. Peroxidase-conjugated Streptavidin (Proteintech) was used for detecting the bound peptide. Absorbance was recorded at 405 nm by a Synergy 2 Multi-Mode Reader (BioTek).

### 2.3. Affinity Measurement by Biolayer Interferometry

Real-time binding assay between peptide MERS-HR2P and MERS-5HB was carried out on an Octet QK system (Fortebio, San Francisco, CA, USA) at 25 °C using biolayer interferometry technology. Specifically, biotin-labeled 500 ng/mL MERS-HR2P was loaded into streptavidin biosensors and then MERS-5HB was added at a series of concentrations: 64 nM, 32 nM, 16 nM, 8 nM, 4 nM, 2 nM. Protein dilutions were dissolved in PBS containing 0.02% Tween (Sangon Biotech), which is better for reducing nonspecific adsorption. Association and dissociation time were both set as 1200 s. The affinity of MERS-HR2P to MERS-5HB was (calculated automatically using the Octet QK software package) (Fortebio) by a 1:1 binding model, and the equilibrium dissociation constant (*K*_D_) value was equal to the kinetic dissociation rate constant divided by the kinetic association rate constant.

### 2.4. Pseudovirus Preparation and Titration

The MERS-CoV pseudovirus was prepared by cotransfecting 293T cells (Shanghai Guidance of Science and Technology, Shanghai, China) (2 × 10^5^ cells/well in a 24-well plate) with 125 ng/well of the plasmid pNL4-3.luc.RE (encoding an Envelope (Env)-defective, luciferase-expressing HIV-1 genome) and 375 ng/well of the plasmid pcDNA3.1-MERS-S (the full-length S gene was cloned from plasmid pUC57-MERS-S kindly provided by Zhengli Shi), using Lipofectamine 3000 Reagent (Invitrogen). Seventy-two hours after transfection, the supernatant containing the pseudovirus was harvested and stored at 4 °C in 1 mL aliquots for short-term use [23,24]. The 50% tissue culture infectious dose (TCID_50_) of the pseudovirus was determined in Huh-7 (Shanghai Guidance of Science and Technology) cells as reported previously [25,26]. Briefly, serial 3-fold dilutions of the pseudovirus were made: 50 μL/well pseudovirus was mixed with 50 μL/well culture medium in quadruplicate in a 96-well white opaque plate (1 × 10^4^ cells/well) with 8 dilution steps in total. The plate was incubated at 37 °C in 5% CO_2_ air environment for 12 h. Then pseudovirus culture was replaced with 100 μL/well fresh medium for an additional 72 h incubation. Bright-Glo Reagent (Promega, Beijing, China), in a quantity of 100 μL, was added directly to cells in 100 μL of culture medium. After 2 min to allow cell lysis, the luminescence was measured with a Synergy 2 Multi-Mode Reader (BioTek).

### 2.5. Inhibition of Pseudotyped MERS-CoV Infection

The assay of MERS-CoV pseudovirus entry inhibition was performed on the basis of previously described methods [10,27]. To detect the inhibitory activity of MERS-5HB, 100 TCID_50_ of MERS-CoV pseudovirus was pre-incubated with 3-fold serially diluted MERS-5HB in quadruplicate from the highest concentration of 15 μM at 37 °C for 1 h. The virus–protein mixture was then transferred to a 96-well white opaque plate seeded with Huh-7 cells (1 × 10^4^/well). Twelve hours later, cells were re-fed with fresh medium (100 μL/well), which was followed by luminescence measurement after a 72-h incubation period. The vesicular stomatitis virus (VSV) pseudovirus was prepared as a control. The inhibition of MERS-CoV pseudovirus was presented as percent inhibition.

### 2.6. Cytotoxicity Assay

The cytotoxicity of MERS-5HB to Huh-7 cells was tested by a Cell Counting Kit-8 (Beyotime, Nantong, China) following the manufacturer’s instruction. MERS-5HB (50 μM) was added to Huh-7 cells (1 × 10^4^/well) in quadruplicate and incubated at 37 °C for 24 h, then the medium was removed and the cells were washed with PBS. Finally, 10 μL of CCK-8 Cell Counting Kit-8 (Beyotime) solution mixed with 100 μL medium was added to each well. Four hours later, the absorbance was measured at 450 nm with a Synergy 2 Multi-Mode Reader (BioTek).

### 2.7. Inhibition of MERS-CoV S Protein-Mediated Cell–Cell Fusion

MERS-CoV S protein-mediated cell–cell fusion was assessed by a modified method [11]. The plasmid pcDNA3.1-MERS-S (500 ng/well) was transfected to 293T cells (293T/MERS-S) and enhanced green fluorescent protein (EGFP) expression plasmid pEGFP-C3 (500 ng/well) was transfected to Huh-7 cells (Huh-7/EGFP) in a 24-well plate, respectively. Six hours later, freshly trypsinized cells of Huh-7/EGFP and 293T/MERS-S were mixed at ratios of 1:1, 3:1, and 9:1. The number of Huh-7/EGFP cells was constant at 1 × 10^5^, while the number of 293T/MERS-S cells was decreased to 1 × 10^5^, 1/3 × 10^5^, and 1/9 × 10^5^. The 293T cells transfected with vector pcDNA3.1 (293T/pcDNA3.1) were used as a negative control. The co-cultured cells were seeded in a 24-well plate, in absence or presence of MERS-5HB, with an initial concentration of 5 μM followed by 3-fold dilutions. After 48 h, the obvious syncytial formation was observed and photographed in light as well as a fluorescence field under an inverted fluorescence microscope Olympus IX71 (Olympus, Tokyo, Japan).

### 2.8. Biophysical Characterization of MERS-5HB

#### 2.8.1. Size Exclusion Chromatography (SEC) Analysis

The purified MERS-5HB was loaded onto a Superdex 75 10/300 GL column (GE Healthcare, Uppsala, Sweden) connected to an ÄKTA purifier chromatography system (GE ÄKTA avant 25) (GE Healthcare) to evaluate its molecular weight (M.W.). PBS was used as the mobile phase with a flow rate of 0.8 mL/min. The ultraviolet absorbance at 280 nm was recorded. A gel-filtration of standard proteins consisting of Bovine serum albumin (67 kDa), β-lactoglobulin (35 kDa), Cytochrome C (13.6 kDa), Aprotinin (6.5 kDa), and Vitamin B12 (1.35 kDa) was used to define the standard curve.

#### 2.8.2. Circular Dichroism Spectroscopy

The secondary structure of MERS-5HB was determined by circular dichroism (CD) spectroscopy. Purified protein was diluted in PBS and adjusted to the final concentration of 1 mg/mL before data collection. The CD spectra from 190 nm to 260 nm were recorded on an Applied Photophysics Chirascan-SF.3 spectrophotometer (Applied Photophysics Ltd, Surrey, UK) at 25 °C in a 0.1 cm path length cuvette (Applied Photophysics Ltd). Thermo-induced unfolding was measured by recording the spectra at 222 nm in temperatures ranging from 25 °C to 95 °C at a ramp-rate of 1 °C/min for evaluation of the thermodynamic stability. The CD data were shown as mean residue ellipticity.

## 3. Results

### 3.1. Design of MERS-5HB

**Figure 1:**
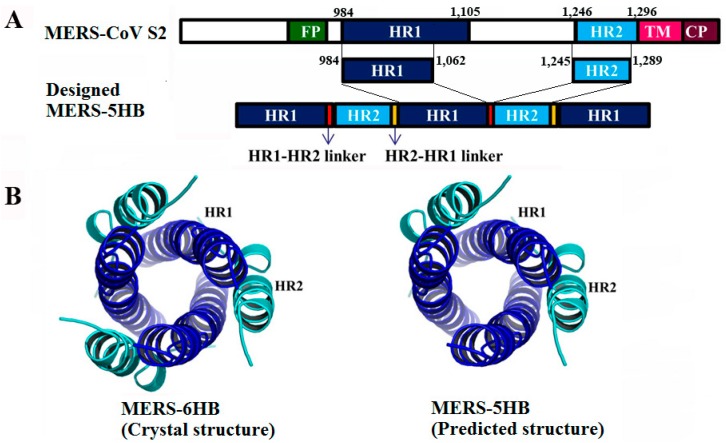
Design of the MERS-five-helix bundle (MERS-5HB) inhibitor. (**A**) Schematic representation of MERS-5HB based on the Middle East respiratory syndrome coronavirus (MERS-CoV) (S2) subunit. Heptad repeat 1 (HR1) (residues 984 to 1062) and heptad repeat 2 (HR2) (residues 1245 to 1289) are from the major parts of HR1 (residues 984 to 1105) and HR2 (residues 1246 to 1296) domains in the S2 subunit. MERS-5HB contains three HR1 segments and two HR2 segments. The linker of HR1 to HR2 is SGGRGG and the linker of HR2 to HR1 is GGSGGSGG. (**B**) Models of the MERS-CoV fusion core (MERS-6HB) (PDB: 4MOD [10]) (**left**) and MERS-5HB (**right**). As a solved crystal structure, the six-helix bundle contains three HR1 segments (shown in blue) which form a central core, and three HR2 segments (shown in cyan) which pack into the HR1 side grooves.

As mentioned in Materials and Methods, the designed MERS-5HB contains three copies of HR1 (residues 984 to 1062) and two copies of HR2 (residues 1245 to 1289), by using flexible SGGRGG and GGSGGSGG as the linkers for connection of HR1 to HR2 and HR2 to HR1 respectively (Figure 1A). Therefore, MERS-5HB could be expressed as a single polypeptide. MERS-6HB exists as a coiled-coil trimer that consists of three copies of HR1 forming a central core, and three copies of HR2 packing into the grooves on the surface of the central core as a solved crystal structure (Figure 1B left) [10]. The predicted structure of MERS-5HB was similar to MERS-6HB although lacking one HR2 (Figure 1B right). The absence of one HR2 may reserve a hydrophobic groove in MERS-5HB for binding to a native HR2 in the MERS-CoV S protein.

### 3.2. Interactions between MERS-5HB and MERS-HR2P

**Figure 2:**
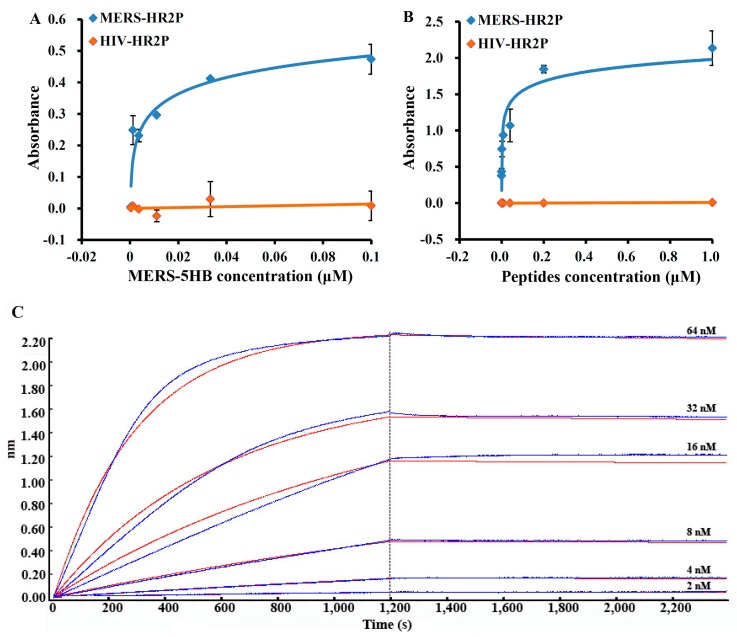
Interaction of MERS-5HB with MERS-HR2P derived from the HR2 region. MERS-HR2P was immobilized on the plate, with MERS-5HB binding detected (**A**). In the reverse, MERS-5HB was coated on the plate with MERS-HR2P for detection (**B**). HIV-HR2P was used as control. All samples were assessed in duplicate and the data were presented as mean ± standard deviation (s.d.) (error bar). (**C**): Binding affinity of MERS-5HB to MERS-HR2P. Peptide was immobilized onto streptavidin biosensors and the binding of MERS-5HB was tested at the following concentrations: 64 nM, 32 nM, 16 nM, 8 nM, 4 nM, 2 nM. The *X*-axis of panel C represents the time of association and dissociation. The *Y*-axis measures wavelength change in biolayer interferometry peaks (in nm), which is a function of changes to the average optical thickness.

The binding between MERS-5HB and MERS-HR2P was tested using ELISA. Firstly, the biotinylated MERS-HR2P was immobilized overnight on a plate coated with streptavidin. Then, MERS-5HB was added with a series of dilutions. The calculated 50% effective concentration (EC_50_) of binding of MERS-5HB to MERS-HR2P is 5.4 nM (Figure 2A). To confirm the binding, the ELISA assay was performed in the reverse way. MERS-5HB was coated onto a plate overnight and MERS-HR2P was added. In this case, the calculated EC_50_ of binding of MERS-HR2P to MERS-5HB is 31.8 nM (Figure 2B). No significant interactions were found between MERS-5HB and HIV-HR2P. In order to accurately determine the binding affinity of MERS-HR2P to MERS-5HB, biolayer interferometry was conducted. The optical sensors were prepared in advance, and consisted of a small length of optical fiber with streptavidin modified on the top. The biotinylated MERS-HR2P was immobilized on the top of the streptavidin biosensors, and the interactions with MERS-5HB at six different concentrations were monitored (Figure 2C). The interaction kinetics result showed that the *K*_D_ value was 2.4 × 10^−10^ M, the association rate constant was 5.26 × 10^4^ ± 1.41 × 10^2^ M^−1^ s^−1^, and the dissociation rate constant was 1.26 × 10^−5^ ± 7.78 × 10^−7^ s^−1^. This demonstrated that the interaction between MERS-5HB and MERS-HR2P is strong and specific, which could make it possible for MERS-5HB to inhibit virus entry as we expected.

### 3.3. Inhibition of Pseudotyped MERS-CoV Infection

**Figure 3:**
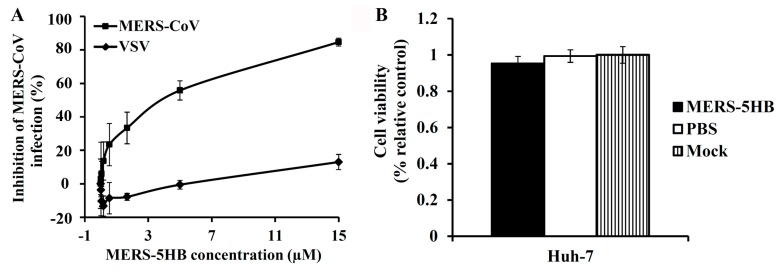
Inhibition of MERS-CoV viral entry by MERS-5HB. (**A**) Inhibitory activity of MERS-5HB on MERS-CoV pseudovirus entry. MERS-5HB was tested for inhibition of a single-cycle infection on Huh-7 cells by pseudotyped MERS-CoV using vesicular stomatitis virus (VSV) as control. The inhibitory effect was described as % inhibition. (**B**) The cytotoxicity of MERS-5HB to Huh-7 cells. PBS was the control and Mock represents normal culture of Huh-7 cells. Experiments were performed in quadruplicate, and the data were presented as mean ± s.d. (error bar). The tests were repeated twice and similar results were obtained.

The inhibitory efficacy of MERS-5HB was evaluated in the pseudotyped virus system. Gradient concentrations of MERS-5HB were incubated with the MERS-CoV pseudovirus, and the inhibitory effect was tested in cultured Huh-7 cells. As shown in Figure 3A, MERS-5HB displayed a notable inhibitory effect for the MERS-CoV pseudovirus but not for the VSV pseudovirus, even at the highest concentration (15 μM), demonstrating the inhibitory specificity of MERS-5HB. The 50% inhibitory concentration (IC_50_) for the inhibition by MERS-5HB of a MERS-CoV infection was about 1 μM, which is comparable to other peptide inhibitors reported previously [10,11]. We then measured the potential cytotoxicity of MERS-5HB to Huh-7 cells which were used for the inhibition assay. No significant cytotoxicity to cell-growth was observed of MERS-5HB at the concentration up to 50 μM (Figure 3B), which proved that MERS-5HB has low or no toxic effect in vitro.

### 3.4. Inhibition of S Protein-Mediated Cell–Cell Fusion

**Figure 4:**
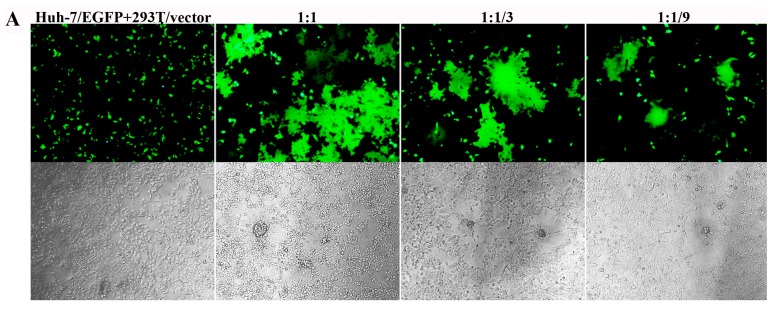

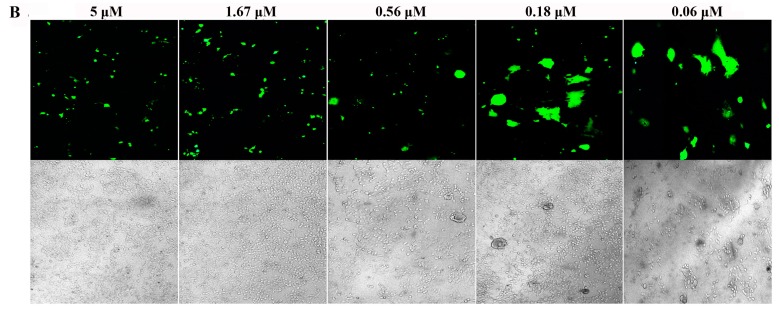
Inhibition by MERS-5HB of MERS-CoV S protein-mediated syncytium formation. (**A**) Images of S protein-mediated cell–cell fusion. Huh-7/EGFP cells and 293T/MERS-S cells were co-cultured at the ratio of 1:1, 3:1, 9:1; 293T/vector cells were tested as control. (**B**) Inhibition of MERS-5HB on cell–cell fusion, with decreasing syncytium formation in the presence of MERS-5HB in a dose-dependent manner. The highest concentration was 5 μM, followed with three-times dilution. The syncytial formation was observed with fluorescence (**top**) or visible light (**bottom**).

To determine whether MERS-5HB was able to inhibit cell–cell fusion mediated by the MERS-CoV S protein, we developed a syncytial formation assay as described in Materials and Methods. Line 293T cells that can instantaneously express MERS-CoV S protein (293T/MERS-S) were co-cultured with Huh-7 cells expressing EGFP (Huh-7/EGFP) as a reporter, using 293T cells with a transfected vector as the control (293T/vector). Forty-eight hours later, effective cell–cell fusion was observed (Figure 4A). The maximum syncytia happened in a proportion of Huh-7/EGFP cells and 293T/MERS-S cells at a ratio of 1:1. In contrast, the syncytial formation was reduced when 293T/MERS-S cells decreased at the ratios of 3:1 and 9:1. In addition, no syncytium was shown when Huh-7/EGFP cells were co-cultured with 293T/vector cells. These results indicated that the MERS-CoV S protein played an important role in cell–cell fusion.

We then tested the inhibitory activity of MERS-5HB in cell–cell fusion. The formation of syncytia was completely inhibited in the presence of 5 μM MERS-5HB and the inhibition effect was dependent on the concentration of MERS-5HB (Figure 4B). The estimated 50% inhibition occurred at a concentration of 0.56 μM, which was consistent with the result of the pseudovirus entry inhibition by MERS-5HB. Because MERS-5HB could effectively bind to the MERS-HR2P representative of viral HR2, it may destroy the native interaction between viral HR1 and HR2, blocking the six-helix bundle fusion core formation.

### 3.5. Biophysical Characterization of MERS-5HB

#### 3.5.1. MERS-5HB Exists as a Monomer

**Figure 5:**
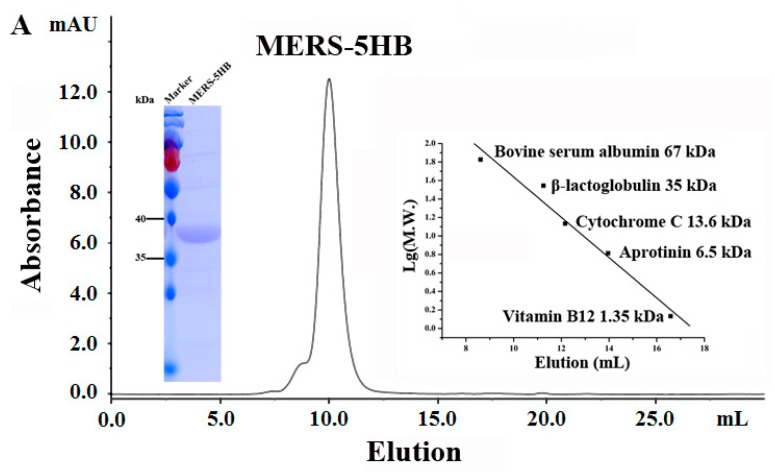

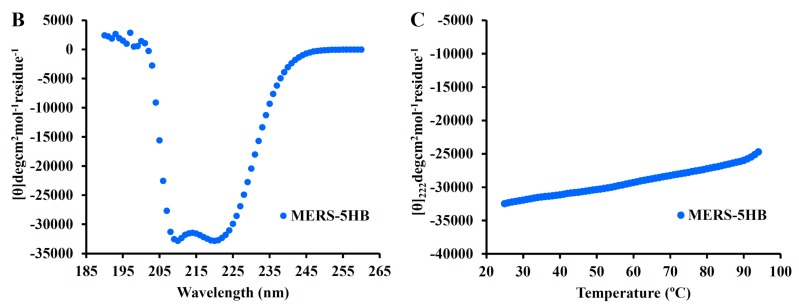
Biophysical characterization of MERS-5HB. (**A**) SEC analysis of MERS-5HB. The elution volume of MERS-5HB was 10 mL indicating that MERS-5HB existed as monomer. (**B**) Secondary structure of MERS-5HB in phosphate buffer, pH 7.4. Circular Dichroism (CD) spectra illustrates double minima at 208 nm and 222 nm indicating that MERS-5HB adopted a typical α-helix structure. (**C**) Thermal denaturation of MERS-5HB. CD signals at 222 nm were recorded from 25 °C to 95 °C with a scanning rate of 1 °C/min.

The M.W. of MERS-5HB in PBS was analyzed using size exclusion chromatography (SEC) analysis (Figure 5A). According to the standard curve, the calculated M.W. of MERS-5HB was 43.4 kDa while the theoretical M.W. of monomeric MERS-5HB was 37.9 kDa, indicating that MERS-5HB existed as a monomer.

#### 3.5.2. MERS-5HB Exhibits Thermo Stable α-Helical Conformation

CD spectra of MERS-5HB at 1 mg/mL in PBS exhibited double negative peaks at 208 nm and 222 nm (Figure 5B), illustrating that MERS-5HB adopts the classic α-helical secondary structure. Thermo-induced unfolding of MERS-5HB was monitored at 222 nm in temperatures ranging from 25 °C to 95 °C. As the temperature increased, the structure was slightly destroyed but with no obvious S- shaped curve appearing (Figure 5C). This helical bundle showed strong thermal stability and appeared to be folded very well, which laid a physiochemical foundation for further study.

## 4. Discussion

MERS-CoV S is an effective target for antiviral agents including prophylactics and therapeutics. The RBD in S1 has already been used for development of neutralizing monoclonal antibodies and vaccines [28,29,30]. However, the virus may mutate in RBD to escape antibody neutralization, and the risk of antibody-dependent enhancement of viral infection also needs to be considered [31,32]. Since S2 is responsible for membrane fusion, it is also an attractive target, especially the fusion core region. In the early 1990s, several synthetic peptides from gp41 in the HIV-1 envelope protein (which is equal to S2 in the MERS-CoV S protein) were identified to inhibit viral entry with high potency through disruption of native interactions between HR1 and HR2 [33,34]. One peptide, T20 (enfuvirtide), is the first HIV-1 entry inhibitor on the market [35]. Following a similar rule, several peptides derived from S2 in the MERS-CoV S protein, as mentioned above, also have been identified as MERS-CoV inhibitors.

Interestingly, besides the short peptide inhibitors from HR2 that could bind to native HR1, a well-designed five-helix protein showed promising activity against HIV-1 through binding to native HR2 but not HR1 [36,37]. Here, we reported that MERS-5HB, designed by us, could bind to HR2 in MERS-CoV S2 to inhibit viral entry as well as S protein-mediated syncytial formation. The inhibition activity is similar to the previously reported short peptide inhibitors [10,11]. Although both MERS-5HB and other peptide inhibitors (e.g., HR2P) block viral entry though disruption of the formation of the fusion core, the detailed mechanisms of their inhibition activities are different. Peptide inhibitors derived from HR2 could interact with HR1 with strong binding force and therefore they are competitors against native HR2. However, due to the absence of one HR2, our MERS-5HB displays high efficacy in binding with HR2 and hence it is a competitor against native HR1. These interesting issues are worthy of being further elucidated.

**Figure 6:**
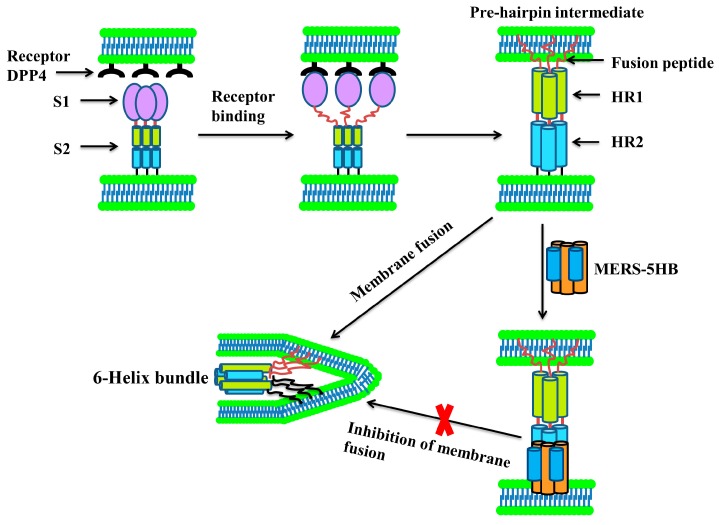
Model of MERS-5HB for inhibition of MERS-CoV entry. One of the key steps for MERS-CoV entry into host cells is the membrane fusion. MERS-5HB, lacking one HR2, could bind to the native viral HR2 in S2 to block the formation of the fusion core which is the central structure during the viral and host membrane fusion process.

After MERS-CoV RBD binding to the receptor DPP4, HR1 and HR2 undergo conformational changes to form a fusion core. In this process, MERS-5HB interacts with native HR2 in MERS-CoV S2, thus blocking the fusion core formation and preventing virus entry (Figure 6). In conclusion, MERS-5HB could be a potential inhibitor against MERS-CoV infection, and it also helps us to throw light upon the mechanism of S protein-mediated membrane fusion.
